# Magnetic Stress Sensing System for Nondestructive Stress Testing of Structural Steel and Steel Truss Components Based on Existing Magnetism

**DOI:** 10.3390/s20144043

**Published:** 2020-07-21

**Authors:** Guangyuan Weng, Jintao Wang, Yang Liu, Xiyu Zhu, Jianbo Dai

**Affiliations:** 1 Mechanical Engineering College, Xi’an Shiyou University, Xi’an 710065, China; 19212040439@stumail.xsyu.edu.cn (J.W.); xiyuzhu@xsyu.edu.cn (X.Z.); jbdai@xsyu.edu.cn (J.D.); 2 School of Urban Planning and Municipal Engineering, Xi’an Polytechnic University, Xi’an 710048, China; yangliu@xpu.edu.cn

**Keywords:** magnetic sensing system, nondestructive test, stress detection, steel structure, existing magnetism

## Abstract

To detect the stress of steel structures and members using the existing magnetism, a magnetic stress sensing system integrating a magnetic flux induction coil, a magnetic flux measurement device, a loaded device, and data acquisition software was developed. The magnetic coupling test research was carried out for different grades of structural building and bridge steel specimens to establish the magnetic stress flux mathematical model, and the fitting equation of the magnetic flux changes with the positions of different sections of specimens was analyzed. Furthermore, a practical formula for stress detection was obtained through the experiments. Meanwhile, on these bases, the typical steel truss structure model of a Bailey beam was designed and manufactured under different working conditions, nondestructive online stress testing was carried out, and the stress of the model structure and its members was measured by strain and magnetic flux tests to obtain the curves of the test results for the stress–strain and magnetic stress flux, respectively. The results of these two methods are in good agreement with each other. The stress of the steel truss model structure was analyzed and calculated using the finite element method. The results agreed well with the experimental results from the magnetic stress sensing system—the maximum error was about 5%, which meets the requirements of engineering applications.

## 1. Introduction

Steel truss structures are some of the most common structural forms in buildings, bridges, and oil and gas pipelines, and are widely used in engineering practice. The key stressed members and parts of the steel truss structures are affected by the changes to the load, material properties, and the environment during construction, operation, and maintenance. If the actual stress state is inconsistent with the design and the service stress reaches or even exceeds the design limit, this can pose a threat to the safety of engineering structures [1,2,3]. Scholars have conducted extensive research on damage identification and health monitoring of such structures [4,5,6,7,8]. Ren et al. took percentages of the identification indices and the identification models they established using C-Support Vector Classification machine (C-SVC) and ε-Support Vector Regression machine (ε-SVR), respectively, to identify 2 bar damage locations and degrees of damage [9]. Bai et al. proposed a structural identification method for a truss structure isolated from damage and identified the damage caused to a practical steel truss bridge [10]. Chen et al. used ANSYS software to perform modal analysis on the spanning structure of an assembled steel truss bridge, and assessed the damage phenomena and vulnerable locations [11]. Yan et al. summarized the research progress in the fields of structural modal parameter identification, damage identification, and model modification [12]. At the same time, scholars have identified the damage caused to different structures according to static and dynamic performance changes, as well as the characteristic parameters of the structures [13,14,15]. Traditional test techniques and methods require information about the loading history of structural elements, which are limited in some respects. 

Under stress, the inverse magnetostrictive effects of ferromagnetic materials change the internal magnetic energy. The stress detection theory based on magnetic coupling effect is a new magnetic nondestructive testing method that was developed in recent years. Scholars have also carried out relevant research on the theory and application of the magnetic coupling effect [16,17,18,19]. Kim and Park fabricated a multichannel magnetic flux leakage (MFL) sensor head using a Hall sensor array and magnetic yokes, which was used to scan damaged specimens to measure the magnetic flux signals [20]. This method will still be complemented and validated by further research performed on various types of damage and environments. Xia et al. proposed a new computing method to quantitatively and nondestructively determine the corrosion of steel strands by analyzing the self-magnetic flux leakage (SMFL) signals from them, which has significant application prospects for the evaluation of corrosion and residual bearing capacity [21]. Shleenkov et al. electrically welded pipes in a technological industrial line using magnetic nondestructive method [22]. Matsumoto et al. proposed a novel magnetic nondestructive testing method to evaluate the residual strain in low carbon steels, relying on the characterization of eddy current signals in the impedance plane when low frequency major magnetization was superimposed [23]. Loskutov et al. summarized a method involving the generation of flaw detectors. A magnetic flaw detector with an advanced design was developed, comprising a cylindrical magnetizing system, impedance fluxgate transducers, and a semiconductor memory unit [24]. 

The research on the theory and application technology of magnetic coupling stress detection started later in China. Yang et al. detected the stress on a steel plate by measuring its coercive force [25]. Zhiyuan et al. built an electromagnetic testing platform based on the magnetic Barkhausen noise (MBN) principle and carried out stress detection research on Q235 steel [26]. Wanqiang et al. detected a gas pipeline using a developed weak magnetic signal acquisition instrument, qualitatively predicted the distribution and depth of defects and the effect of stress on the magnetic signal and found the perforation position of a buried gas pipeline [27]. Xiong et al. established a correlation between the magnetic flux and magnetic induction intensity of Q235 steel members and the stress caused by external load through experiments [28]. Xihua et al. proposed a weak magnetic stress internal detection technology for online stress detection and risk assessment of oil and gas pipelines [29]. In addition, relevant scholars have established and applied magnetomechanical models of different structures by using the theories and techniques of electromagnetism, magnetism, and magnetoelasticity [30,31,32,33]. Overall, in the fields of magnetic properties and nondestructive testing, scholars have carried out some research on the theory of magnetic sensors and damage detection methods for steel bridges, building structures, and pipelines. However, the stress of a structure or structural member in service is difficult to detect directly; therefore, further theoretical and experimental research is still needed in terms of magnetic parameter–stress models and applications.

In order to overcome the deficiencies of the loading history information required in traditional stress damage detection of steel truss structures, this paper explores a magnetic sensing system and its application method of nondestructive online stress testing for common steel truss structures by using the magnetomechanical properties of ferromagnetic materials. In the case of no excitation on the specimens, the paper studies the magnetic stress flux mathematical model based on the existing magnetism of structural building steel and structural bridge steel. Model structure simulation test and numerical simulation analysis are carried out, and a magnetic stress sensing system and a more effective nondestructive online stress detection method are proposed. The magnetic coupling model under the excitation of an external magnetic field needs to consider the influences of excitation, magnetic measurement, and other factors, which make its application too. Therefore, the magnetic stress flux model based on existing magnetism proposed in this paper can simplify the method of magnetic nondestructive stress detection, which will enrich the existing achievements of damage identification and magnetic nondestructive online stress detection theory.

## 2. Theoretical Background

### 2.1. Structural Steel and Its Magnetic Properties

The structural steels studied in this paper are structural building steel and structural bridge steel. In the material analysis, structural building steel specimens (Q235B, Q345B, Q390B, Q420B) and structural bridge steel specimens (Q235qD, Q345qD, Q375qD, Q420qD) were made to analyze the magnetic flux based on existing magnetism. By analyzing the theoretical basis of the magnetic mechanics, the design method for the nondestructive magnetic coupling stress testing probe used to measure the magnetic flux was found, the fitting equation for the magnetic stress coupling was proposed, and the magnetomechanical test and simulation calculation verified the validity. The results of the magnetomechanical properties test and the magnetic coupling stress test in this paper were based on the material composition and mechanical properties shown in Table 1. The chemical composition and mechanical properties of the structural steel in Table 1 are representative; therefore, the test results in this paper are applicable to the stress testing of general structural steel. For some special structural steels, the corresponding test results can also be obtained by referring to the research method in this paper.

**Figure 1:**
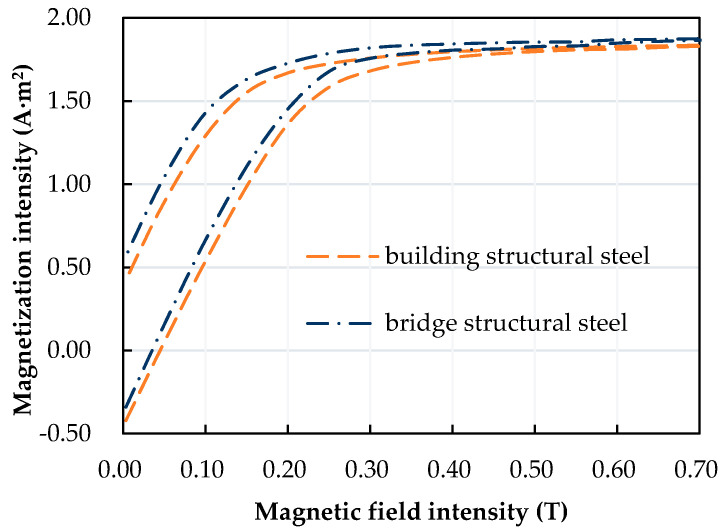
Magnetization curves of Q420B structural building steel and Q420qD bridge steel.

Generally, the magnetization curve can describe the magnetic characteristic parameters of ferromagnetic materials. The magnetization curves for Q420B structural building steel and Q420qD structural bridge steel are shown in Figure 1.

In Figure 1, The X axis represents the strength of the magnetic field. We use a method to adjust the magnitude of the current in the coil to control the strength of the magnetic field. After preliminary calculation, the strength of the magnetic field was controlled within the range of 0–0.7 T. The Y axis represents the magnetization of the material. By applying external magnetic fields of different strengths, the magnetization of the material can be measured by the magnetic measurement device mentioned in this paper.

As seen in Figure 1, for the convenience of analysis, the part of the magnetization curve with a positive magnetic field was used. Structural building steel and structural bridge steel have similar magnetization curves, which gives a widely applicable theoretical basis for developing stress sensing probes based on the magnetic stress coupling effect. In the magnetization stage, the magnetic domain structure movement tends to be consistent with the direction of the magnetic field and the magnetization intensity of the specimens increases. When the magnetic field intensity reaches 0.7 T, the magnetization intensity no longer increases and reaches a stable saturated magnetization state. Under the same magnetic field intensity, the saturation value of the magnetization intensity of the structural bridge steel is slightly higher than that of the structural building steel, which can reach 1.87 A·m^2^.

Different brands of materials have different production processes. For the different grain structures of round steel specimens, although they have similarly shaped magnetization curves, the maximum magnetization intensities are different. The maximum magnetization is an extremely important magnetic parameter of ferromagnetic materials. According to electromagnetic induction, measuring the magnetic flux in or around the sample can determine the maximum magnetization. In this paper, an induction coil probe was used to measure the magnetic flux of the structural steel samples in the existing magnetic field, thereby establishing the relationship between magnetic flux and stress. Therefore, the maximum magnetization is an important parameter for magnetic induction stress detection, which has an important influence on the design and manufacture of magnetic induction probes. The maximum magnetization intensity values of specimens with different grades are shown in Table 2, which provides a basis for exploring the constitutive model of magnetic stress flux based on existing magnetism.

From Table 2, it is known that the maximum magnetization intensity of the structural bridge steel with the same grade is higher than that of the similar structural building steel.

### 2.2. Magnetic Stress Sensing Theories

According to previous research [34,35,36], the study of the magnetomechanical properties of structural steel was carried. Structural steel has certain residual magnetism and weak magnetism in smelting, forming, processing and technical magnetization. This weak magnetism changes under the action of geomagnetic fields, which is called the existing magnetism of structural steel. When the structural steel specimens are subjected to force, the stress causes changes of the domain structure movement. This causes the magnetic flux of the structural steel specimens based on the existing magnetism to change, which can be used to detect stress on the structural steel members. The existing magnetic field distribution of the structural steel specimens can be expressed by the physical magnetic flux. The coupled effect of the magnetic field and stress changes the magnetization curve of the structural steel specimen— when the tensile force is applied the magnetization increases, and when pressure is applied the magnetization decreases. Regardless of the existence of external or existing magnetic fields, as long as the magnetization is not zero the effect of stress on the magnetization curve is the same.

For ferromagnetic materials such as structural steel, the energy of the ferromagnetic cubic crystals related to the direction of the saturation magnetization can be expressed as

(1) $$\begin{array}{c} W_{m}=K_{1}\left(\alpha_{1}^{2}\alpha_{2}^{2}+\alpha_{2}^{2}\alpha_{3}^{2}+\alpha_{1}^{2}\alpha_{3}^{2}\right)+W_{me}\\ =K_{1}\left(\alpha_{1}^{2}\alpha_{2}^{2}+\alpha_{2}^{2}\alpha_{3}^{2}+\alpha_{1}^{2}\alpha_{3}^{2}\right)-\frac{3}{2}\lambda_{\left(100\right)}\sigma \left(\alpha_{1}^{2}\gamma_{1}^{2}+\alpha_{2}^{2}\lambda_{2}^{2}+\alpha_{3}^{2}\lambda_{3}^{2}\right)\\ -3\lambda_{\left(111\right)}\sigma \left(\alpha_{1}\alpha_{2}\gamma_{1}\gamma_{2}+\alpha_{2}\alpha_{3}\gamma_{2}\gamma_{3}+\alpha_{1}\alpha_{3}\gamma_{1}\gamma_{3}\right) \end{array}$$

where $W_{m}$ is the magnetostrictive energy; *K*_1_ is magnetocrystalline anisotropy constant; $\alpha_{1},\;\alpha_{2},\;and\;\alpha_{3}$ are the direction cosines of the spontaneous magnetization; $W_{me}$ is the magnetoelastic energy related to the magnetostrictive deformation and stress; σ is stress; $\gamma_{1},\gamma_{2},\gamma_{3}$ are the direction cosines of σ; $\lambda_{\left(100\right)},\lambda_{\left(111\right)}$ are the magnetostriction coefficients along the crystal axis.

If the magnetostriction of the ferromagnetic material is isotropic, $\lambda_{\left(100\right)}=\lambda_{\left(111\right)}=\lambda_{s}$, the magnetoelastic energy can be expressed as

(2) $$W_{me}=-\frac{3}{2}\lambda_{s}\sigma \cos^{2}\theta$$

where θ is the angle between the spontaneous magnetization and the stress, which affects the magnitude of the magnetoelastic energy. If we replace $\cos^{2}\theta$ with $\left(1-\sin^{2}\theta \right)$ and discard the term that does not contain θ as a constant, then

(3) $$W_{me}=\frac{3}{2}\lambda_{s}\sigma \sin^{2}\theta$$

It can be seen from Formulas (2) and (3) that the responses of the ferromagnetic materials to stress vary with the positive or negative values of $\lambda_{s}$ and σ. Positive values of $\lambda_{s}$ loaded by tensile (σ>0) are the same as negative values of $\lambda_{s}$ loaded by compression (σ<0). Under the described circumstances, the magnetized material will be elongated. The magnetization increases with tension and decreases with pressure [37].

**Figure 2:**
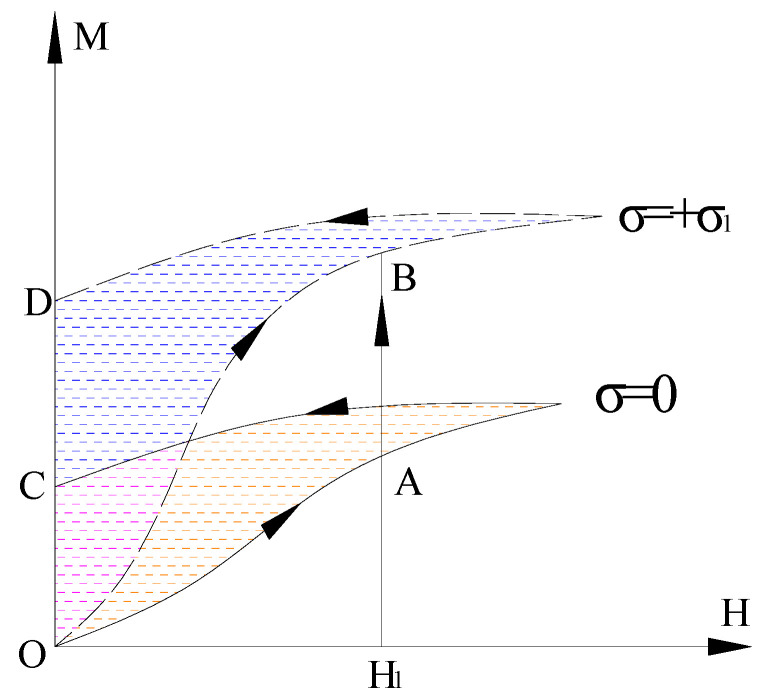
Schematic Diagram of a magnetization curve under tensile stress.

When the structural steel sample is subjected to tensile stress and the magnetostriction coefficient is positive, a magnetic field can be applied in the direction parallel to the tensile stress of the structural steel sample, leading to the behavior shown in Figure 2. Due to the positive magnetostriction coefficient values of the samples [38], the magnetic flux of the structural sample is employed in this paper to study the stress detection by analyzing the mechanism of the changes in tensile stress and magnetic properties of tensile structural steel specimens under the principles shown in Figure 2.

It can be seen from Figure 2 that without stress, the magnetization generated by the magnetic field strength *H*_1_ is A. Under the same magnetic field, if stress $+\sigma_{1}$ (tensile stress, for example $\sigma_{1}=+200\;\mathrm{MPa}$) is applied, the magnetization will increase to point B. The residual magnetization without stress is C and the residual magnetization increases to point D under tensile stress. By using the principle that stress action can change the magnetization of structural steel, in this paper magnetic sensing for stress detection of steel truss components is developed.

### 2.3. Magnetic Flux Sensing System

**Figure 3:**
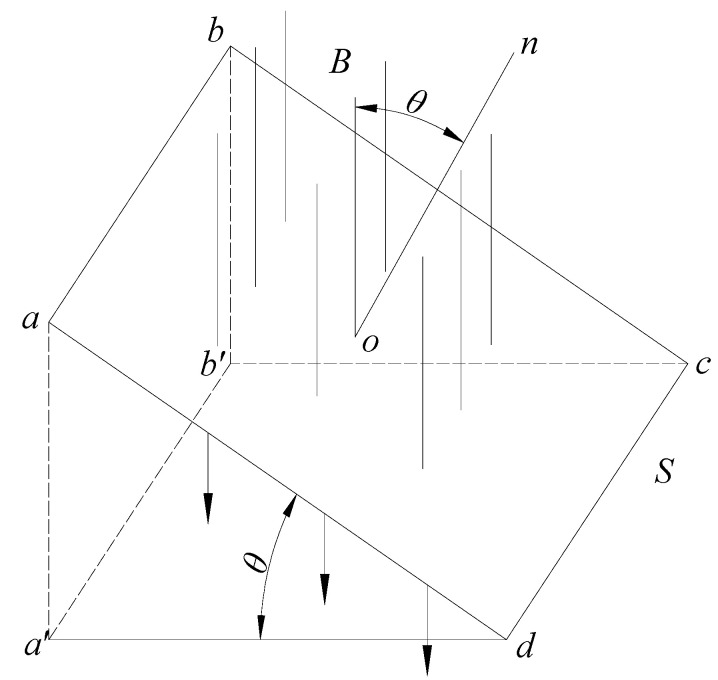
Magnetic flux distribution of a cross-section.

In research, magnetization intensity is a key factor for the working principle used for magnetic sensing probes. However, in addition to this, the geometric characteristics of the component to be tested are also key factors. The magnetic flux can influence the cross-sectional characteristics and magnetization intensity of the measured components. In this paper, the magnetic flux based on the existing magnetism is taken as an important parameter of the relationship between the magnetic flux and stress changes of structural steel samples with different cross-sections. The relationship between the cross-sectional magnetic flux density and the cross-sectional position can be described using Figure 3.

As shown in Figure 3, B is the magnetic induction intensity, S (surface a-b-c-d) is the surface, and *θ* is the angle between the normal direction *n* of the panel and the magnetic induction density B. Magnetic flux is defined by the integral of the magnetic field on the surface area, represented by Φ. The magnetic flux of dS passing through a surface element in magnetic field B is dΦ = BdScosθ. The projection of dS perpendicular to B is dScosθ. The magnetic flux value passing through an arbitrary closed surface is determined by the algebraic sum of the magnetic flux of the infinitesimal surface element. For the closed surface, the outer normal vector is positive when θ < 90° and negative when θ > 90°. The cross-sections of the steel truss model elements tested in this paper are all perpendicular to the direction of magnetic induction intensity; that is, θ = 90°. In the actual test, the magnetic flux is obtained by measuring the voltage change of the coil and then calculated by an electronic integrator.

In this study, a sleeve-type magnetic–stress sensing system is used and the magnetic flux induction device of the component to be measured is implemented by an energized measurement coil. The structural design of the measurement coil and the measurement effect have significant influence. Therefore, it is necessary to study the formation and distribution of magnetic induction in the magnetic flux measurement coil. A properly designed magnetic flux measurement coil should form a nearly uniform magnetic induction field inside the coil where the component to be measured is located.

The ideal electromagnetic coil refers to a coil whose length is infinite, and the wire is wound in a single layer. When the current *I* passes through the coil, the magnetic induction in the coil can be expressed as

(4) $$B_{0}=\mu_{0}nI$$

where $B_{0}$ is the magnetic induction of the ideal solenoid coil, $\mu_{0}$ is the vacuum permeability, *n* is the number of coil turns, and *I* is the current intensity.

In practice, the magnetic flux induction measuring coil is often a multilayer coil. The magnetic field distribution in the axial direction of the multilayer coil can be regarded as being formed by stacking multiple single-layer coils with the same length but different aspect ratios. For a multilayer magnetic flux induction coil, the magnetic induction intensity *B_x_* located *x* along the axial direction is

(5) $$B_{x}=\frac{\mu_{0}nI}{2∆r}\left[x\ln\frac{R_{0}+\sqrt{R_{0}^{2}+x^{2}}}{R_{i}+\sqrt{R_{i}^{2}+x^{2}}}+\left(l-z\right)\ln\frac{R_{0}+\sqrt{R_{0}^{2}+{\left(l-x\right)}^{2}}}{R_{i}+\sqrt{R_{i}^{2}+{\left(l-x\right)}^{2}}}\right]$$

where $B_{x}$ is the magnetic induction of the multilayer coil; *R*_0_ and *R_i_* are the inner diameter and outer diameter of the coil, respectively; ∆r is the layer thickness. the ideal layer thickness is $∆r=\frac{Nd^{2}}{l}$, and if layer gaps are considered, $∆r=\frac{1.2Nd^{2}}{l}$.

**Figure 4:**
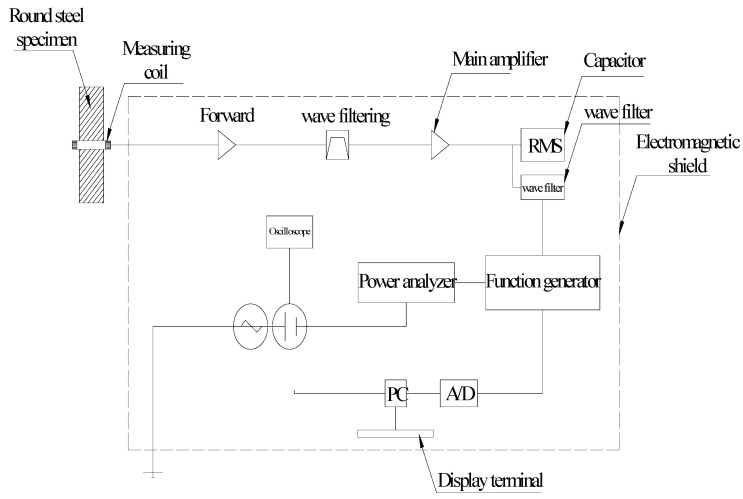
Main components of the magnetic flux sensing system.

In this study, a finite-length multilayer electromagnetic coil was used as a magnetic stress sensing probe. The diameter and length of the coil were determined according to the cross-sectional characteristics of the measured component and the number of coil layers was determined according to Formula (5). After repeated calculation and test verification, the designed magnetic stress sensing probe was designed and made. Based on existing magnetism, a magnetic flux measurement system of the magnetomechanical effect was developed, including a magnetic stress sensing probe, magnetic flux acquisition element, display unit, control circuit, integrator, control unit, and more. The principle diagram of the magnetic flux measurement system is shown in Figure 4. The magnetic flux acquisition element involved a fluxmeter with a measuring coil. The winding inner diameter of the sensing coil was customized according to different diameters of specimens. The five common measuring coils with inner diameters of 12, 16, 20, 25, and 32 mm were customized in the study. The winding component was made of 0.18 mm copper wire with 500 winding turns.

### 2.4. Existing Magnetism of Structural Steel Specimens Measured by Magnetic Sensing Coil

According to the actual materials used in the project, round steel specimens of structural steel were selected. The diameters of the round steel were 12, 16, 20, 25, 28, and 32 mm. In this test, round steel specimens with different diameters; grade Q235B, Q345B, Q390B, and Q420B structural building steels; and grade Q235qD, Q345qD, Q375qD, and Q420qD structural bridge steels were made, numbering 120 specimens in total. In order to eliminate the influence of the end restraint of the specimens, the length of the specimens with diameters of 12, 16, 20, 25, and 28 mm were 400 mm, and that of specimens with diameters of 32 mm was 500 mm.

**Figure 5:**
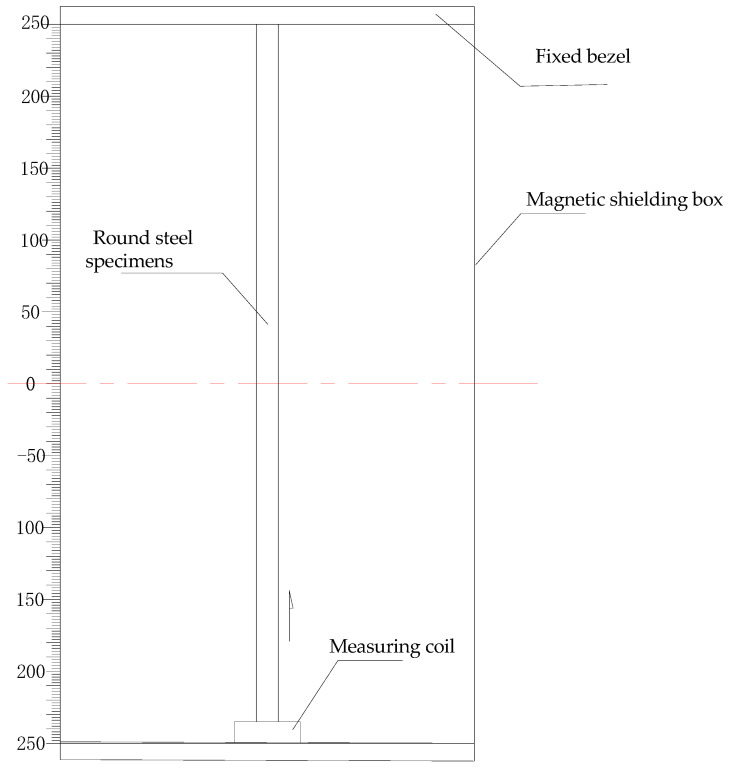
Magnetic flux measurement principle for steel specimens.

It is very important to shield the magnetic interference from the surrounding electromagnetic environment. In order to reduce environmental electromagnetic interference, ferronickel soft magnetic alloy 1J85 permalloy was selected as the shielding material to make the magnetic shielding box. A shielding box can shield the interference of the external magnetic field and keep the box in a relatively stable magnetic field. After the actual test of the magnetic field in the box, as shown in Figure 4, the magnetic field in the middle part (0 mm) of the box was the largest, while the shielding of the magnetic field at the top (250 mm) and bottom (−250 mm) was the best, where there was almost no magnetic field nearby. Taking the circular steel specimen with diameter of 32 mm as an example, the principle of the magnetic flux measurement based on existing magnetism is shown in Figure 5.

The process of measuring the magnetic flux of the existing magnetism of structural steel specimens was affected by temperature, humidity, and external magnetic field interference conditions, which have great effects on the magnetism. The specimens were placed in the magnetic shielding box. The changes of magnetic flux in different positions of the specimens were tested in the magnetic field of the box. The test was divided into forward and reverse tests. The forward test was first used to place and zero the fluxmeter. Then, the magnetic flux value was measured every 50 mm along the length of the round steel from bottom to top. The measurement range was from −250 mm to 250 mm. The reverse test was used to clear and record the fluxmeter after the forward test was completed and the round steel specimens were installed upside down. Then, the coil was moved slowly and uniformly from the bottom to the top along the length of the round steel and the magnetic flux value was measured every 50 mm. For this test, the environment with little interference from the surrounding magnetic field was selected to maintain constant temperature and humidity, while the influence of self-weight was ignored.

**Figure 6:**
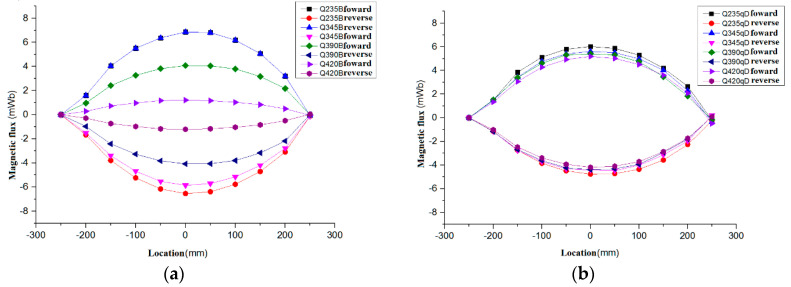
Magnetic flux of structural steels with a diameter of 32 mm. (**a**) Magnetic flux of building structural steel specimen, (**b**) Magnetic flux of Bridge structural steel specimen.

Figure 6 shows the distribution diagram based on the magnetic flux of the existing magnetism, along with the round steel specimens of different grades, with a 32 mm diameter and specimen lengths.

It can be seen from the comparison of the test results that the magnetic flux test values for Q235B based on existing magnetism were the largest, while the magnetic flux test values of Q420B were smallest. From the test results, it is known that the forward and reverse data measured are symmetrically distributed on the axis where the magnetic flux is 0, forming a closed curve. Because the two ends of the sample were close to the top and bottom of the shielding box, the magnetic field at this position was almost zero. When the measuring coil was located at the top (250 mm) or the bottom (−250 mm), the magnetic induction intensity B was zero under the influence of the shielded magnetic box. Therefore, the magnetic flux at both ends in Figure 5 is zero. In the process of the coil moving from the end to the middle part, the magnetic flux increased with the increase of the magnetic induction intensity. In addition, it should be noted that the magnetic flux curves obtained by Q235B and Q345B forward measurement are almost coincident in Figure 5. These results only have a certain relationship with the measurement operation and do not mean that there is an equal amount. In the subsequent experiments, we confirmed that the error of this operation is negligible for engineering applications.

As shown in Figure 6, for the D-class series of structural bridge steels, the magnetic flux decreases with the grades from Q235qD, Q345qD, Q375qD, to Q420qD. Compared with the structural building steel, the curve of magnetic flux change with the location is relatively dense. The magnetic flux of structural bridge steel of the same grade based on existing magnetism is larger than that of structural building steel, and the magnetic flux is less affected by grade.

The curve in Figure 6 is fitted numerically to obtain the fitting equation of the magnetic flux along the length of the specimen, as shown in Table 3.

In the magnetic flux test based on existing magnetism, the influence of stress on the movement of the magnetic domain structure under the action of dead weight is ignored. The established magnetic flux fitting model can be used as the existing magnetic model under a zero stress state. In order to further study the magnetic stress flux model based on existing magnetism of structural building steel and structural bridge steel specimens under different stress states, numerical analysis and experimental study were adopted to reveal the law of change for the magnetic flux based on the existing magnetism of the specimens. This provided a reliable theoretical basis for online nondestructive stress detection based on existing magnetism.

### 2.5. Magnetic Stress-Sensing System and Application for Structural Steel Specimens

**Figure 7:**
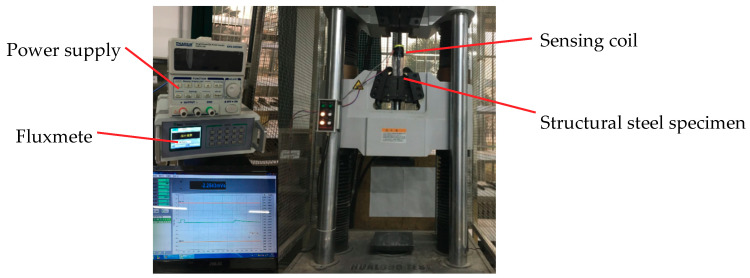
Effect testing of Tensile Force on Magnetic Flux.

This section mainly studies the relationship between the magnetic field and tensile stress generated by the existing magnetism of structural steel specimens. In the elastic range, the relationship between the tensile stress and the change of the existing magnetic field of specimens is established by experimental analysis. Through the self-developed stress measurement system for the magnetic coupling effect, using a WAW-1000 microcomputer-controlled electrohydraulic servo universal testing machine, loading and unloading were performed at the rate of 10 MPa/s, and the cycle was repeated five times. Figure 7 shows the magnetic flux test of structural steel specimens based on existing magnetism under different tensile forces.

**Figure 8:**
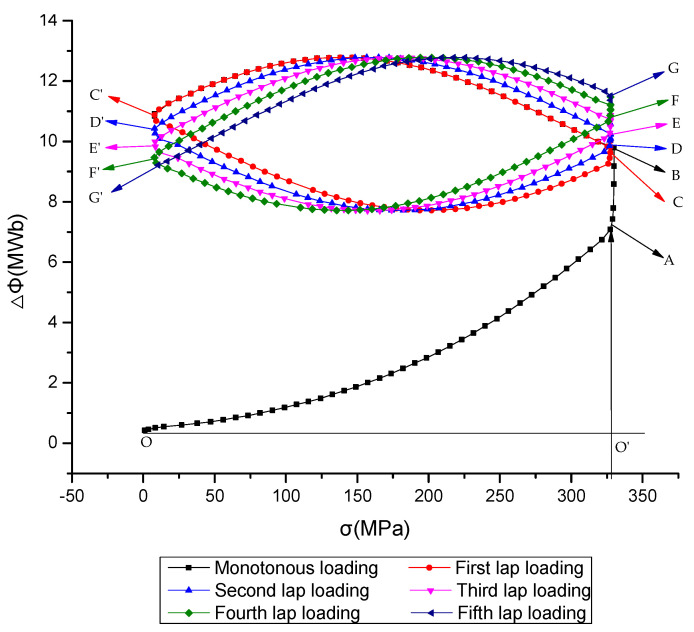
Magnetic stress flux curve of structural steels with diameters of 32 mm.

Seventy-two specimens of different structural bridge steels (grades Q235qD, Q345qD, Q375qD, and Q420qD) and structural building steels (grades Q235B, Q345B, Q390B, and Q420B) were selected for tensile tests. The stress–time curve and magnetic flux–time curve were obtained by measuring the flux variation of the middle section. The magnetic stress flux coupling treatment was carried out to establish the magnetic flux–stress curve based on the existing magnetism of the structural steel specimens. Taking Q390B structural building steel and Q375qD structural bridge steel with diameters of 32 mm as examples, the magnetic stress flux curves based on existing magnetism are given in Figure 8.

The principle of magnetic stress testing studied in this paper is within the range of elastic stress; that is, the curve of the O-A segment in Figure 8. Point A is the magnetic flux corresponding to the optimal magnetic elastic state of the structural steel specimen. Before loading the specimens to the proportional ultimate strength at point A, the tensile stress is about 325 MPa. The magnetic flux of the test section increases with the increase of tensile stress, meaning the magnetic flux is about 7.0 MWb, while the increase rate is lower than that of the tensile stress. When the tensile stress reaches the proportional ultimate strength, the elastic deformation of the specimen shrinks the cross-section, then the magnetic flux increases linearly and reaches the saturation state at point B, where the magnetic flux is about 9.7 MWb. Subsequently, the tension stress of the specimen decreases gradually and uniformly after unloading, giving the curve C–C’. When the elastic recovery deformation makes the cross-section area increase slightly, the co-directional motion of the magnetic domain structure makes the magnetic induction intensity increase. This results in a slightly increased magnetic flux curve with the decrease of the stress to about 2.2 MWb. After this, the specimen section increases continuously under the elastic recovery deformation and the magnetic flux curve shows a descending section of about 1.7 MWb. The first loading and unloading processes are completed as C–C’. In the the second to fifth loading processes, the magnetic flux curve has the same change law. In Figure 8, the curves C–C’, D–D’, E–E’, F–F’, and G–G’, respectively, represent the first to fifth loading and unloading processes. The magnetic flux decreases slightly in the process of loading and then increases slightly. In the process of unloading, the magnetic flux first increases slightly and then decreases slightly. This change is related to the area increase or shrinkage of the tested cross-section, the movement of the magnetic domain structure, the influence of the test coil, and the change of the magnetic induction intensity. In this paper, the constitutive model of the stress flux in the first monotonic loading process is studied. The results of the tension or magnetic flux measurements of 72 specimens show that the magnetic stress flux curves of structural steel specimens with different grades and diameters are similar, and a unified constitutive magnetic stress flux model can be established in the linear elastic range.

According to the magnetic flux and tensile stress data measured by monotonic loading for the first time in this test, the fitting relationship between flux variation and stress was analyzed and the constitutive magnetic stress flux model was obtained.

By fitting the magnetic flux data obtained from the first elastic loading at the initial stage, the constitutive equation of the magnetic flux change and tensile stress of the selected round steel specimens of structural building steel and structural bridge steel were obtained.

(6) $$\begin{array}{c} \sigma =a-b\times \ln\left(\Delta \Phi \right)\\ a=32.83+0.5208\times f_{\mathrm{yk}}-7.289\times d-0.0077\times f_{\mathrm{yk}}\times d+0.1639d\\ b=0.9526-0.3064\times f_{\mathrm{yk}}+0.0002\times d \end{array}$$

where σ is the stress value in MPa; ΔΦ is the variation of the magnetic flux in mWb; *a* and *b* are the parameters determined by the grade and diameter of the round steel, which are obtained by regression analysis of the test data; *f*_yk_ is the standard value of the yield strength of buildings and bridges in MPa; and *d* is the diameter of specimen in mm.

Formula (6) shows that under the action of tensile stress, the change of magnetic flux based on the existing magnetism of structural steel specimens is related to the diameter and grade of the specimen. On this basis, the database program for the magnetic flux variation of the embedded structural steel specimens and the coupling curve of tension stress was developed using MATLAB. Similarly, a database program for the coupling curves of compressive stress and magnetic flux was established, which provides a theoretical model and experimental basis for the magnetic coupling stress detection of tensile specimens.

## 3. Experimental Study

### 3.1. Steel Truss Model Structure

Taking a 24 m steel truss spanning structure supporting an oil and gas pipeline as the prototype, the test model was made at a scale of 1:20.

Considering the equipment and load-carrying capacity used in the static loading test, the material chosen for the model was structural building steel or structural bridge steel. The load-carrying capacity was basically the same as that of the prototype. According to the similarity theory, the corresponding dimensions were analyzed and the similarity between the calculation model and the prototype structure were carried out. In the test, the stress state of the rod under static load was tested using the traditional strain gauge and the magnetic flux measurement method based on the existing magnetism at the same time, and the reliability of these two methods was compared.

**Figure 9:**
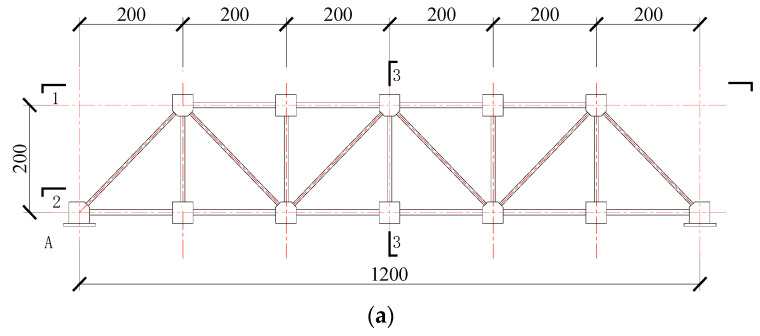

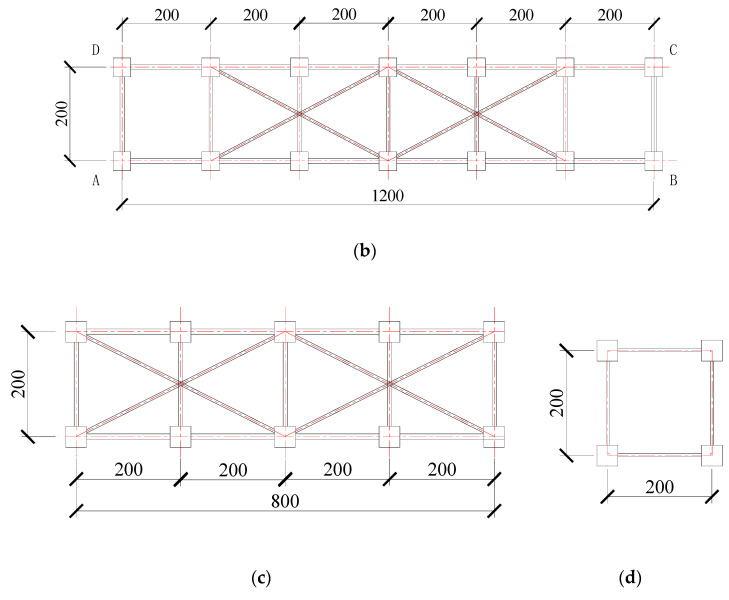
Design of the steel truss model structure (mm): (**a**) steel truss model; (**b**) 1-1 plane sketch of the bottom beam; (**c**) 2-2; (**d**) 3-3.

In order to study the application of the constitutive stress flux model based on the existing magnetism in the stress detection of the main stress members of the steel truss structure, a test model of the steel truss structure was designed, taking the common Berley beam steel truss structure as the prototype. The model structure design is shown in Figure 9.

Steel truss model joints and rods were bolted to facilitate the replacement of different grades of structural building steels or structural bridge steels. The steel truss model structure consisted of 62 rods, including 17 upper chords, 23 lower chords, and 22 web (oblique) members. All the links between the rods had cold connections, which were convenient for disassembling and installing test instruments in the test. In this experiment, the stress and magnetic flux in the middle section of test rod were tested.

### 3.2. Magnetic Stress Sensing System for Steel Truss Structure

**Figure 10:**
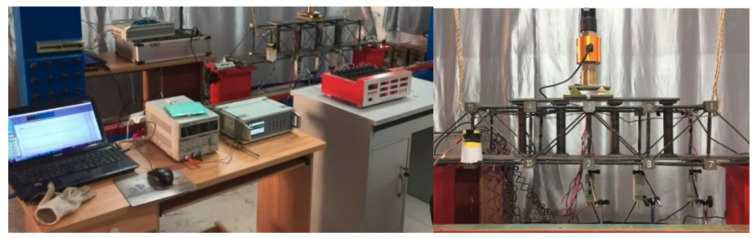
Magnetic stress sensing system for steel truss structures.

The magnetic sensing coil discussed in the previous section was still the core component of the magnetic stress sensing system for stress detection of the steel truss model structure based on the existing magnetic. The counterforce frame with multichannel data transmission function is an important element that can apply force to the steel truss model structure, and is also one of component of the magnetic stress sensing system used for the stress testing. The loading process simulates the stress state of the steel structure in service. The model loading and test acquisition equipment are shown in Figure 10.

**Figure 11:**
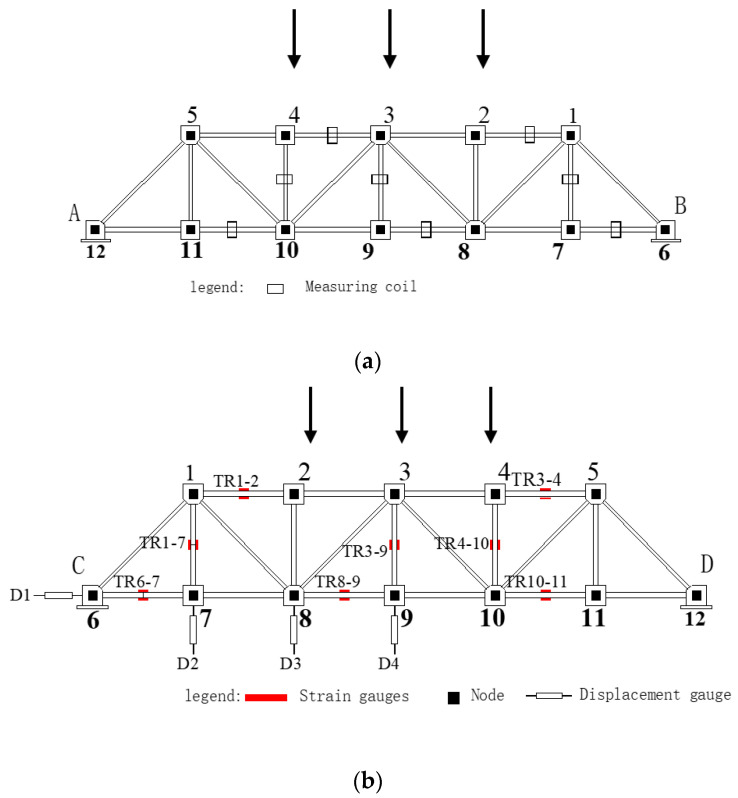
Test point arrangement for stress detection testing of steel truss components: (**a**) A–B plane truss; (**b**) C–D plane truss.

In the experiment, every member of the steel truss model structure was loaded within the linear elastic range in steps of 5kN, and the loading speed was controlled at 0.15–0.25N mm^−2^·s^−1^). The load in this test can be considered as a static load. The YHD-50 displacement sensor was selected and the XL2118A static resistance strain gauge was used to collect test data, which was compared with that collected by the magnetic stress sensing system to verify the correctness of the stress testing method this paper developed. The face direction in Figure 10 is defined as the AB plane of the model structure and the relative direction as the CD plane. The layout of the specific measuring points is shown in Figure 11.

In the static load test of the steel truss model structure, in order to reduce the mutual interference between the test devices used in these two test methods, displacement meters and magnetic flux test devices were arranged for different members in the same stress position using the symmetry of the model structure. In the CD plane of the steel truss shown in Figure 11b, eight groups of strain gauges were set, respectively, with specific positions of sections 1–2, 1–7, 3–4, 3–9, 4–10, 6–7, 8-9, and 10–11. The strain gauge was set up to test the stress variation of the corresponding members of steel truss model structures under different loads. At the same time, vertical displacement meters were set up at 7 (1/6 spans), 8 (1/3 spans), and 9 (mid-span) joints of the CD plane to test and record the deflection changes of the overall steel truss model structure in order to determine or adjust the loading conditions. The magnetic flux measurement coils based on the existing magnetism developed in this paper were arranged in the middle of sections 1–2, 1–7, 3–4, 3–9, 4–10, 6–7, 8–9, and 10–11 of the AB planes shown in Figure 11a. In the experiment, the axial tension stress of the member was determined by measuring the displacement gauge and magnetic flux change in the loading. The results were compared and analyzed.

### 3.3. Test Results

**Figure 12:**
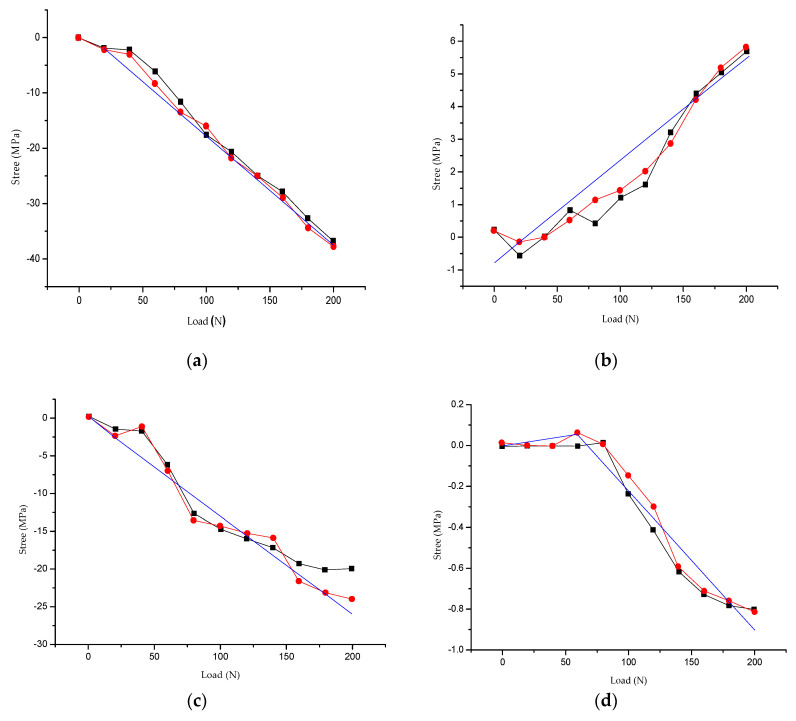

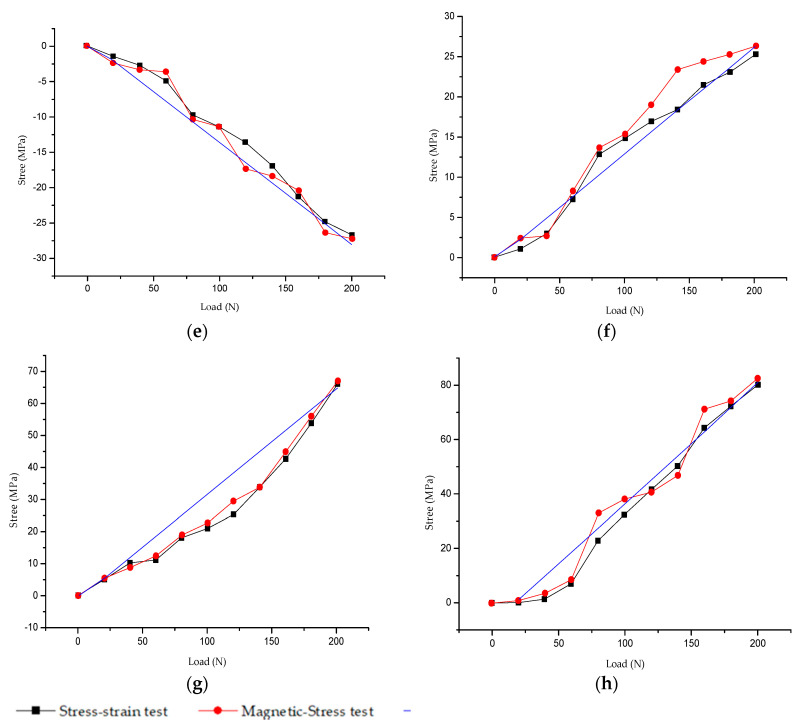
Comparisons of numerical calculations and tests: (**a**) 1–2 rod, (**b**) 1–7 rod, (**c**) 3–4 rod, (**d**) 3–9, (**e**) 4–10 rod, (**f**) 6–7 rod, (g) 8–9 rod, (**h**) 10–11 rod.

In the static test of the steel truss structure model, the magnetic flux measurement system based on the existing magnetism was used to measure the variation of the magnetic flux in the middle positions of sections 1–2, 1–7, 3–4, 3–9, 4–10, 6–7, 8–9, and 10–11 of the AB plane. The constitutive stress flux model was used to obtain the stress curve. Corresponding to the AB plane, the CD plane of the steel truss was measured using a traditional strain gauge, while the strains in the middle positions of sections 1–2, 1–7, 3–4, 3–9, 4–10, 6–7, 8–9, and 10–11 were obtained in the process of loading. The stress–strain curves were obtained using the stress–strain relationship. Taking the steel truss model structure of the Q420qD structural bridge steel as an example, this paper presents the results of the two test methods used in the same positions of the middle sections of the members. After analysis, the load–stress curve was obtained. The results are shown in Figure 12.

The magnetic flux measured by the magnetic–mechanical effect system was calibrated. In the same test environment, the magnetic flux of the tested object was measured when the model structure was not loaded, while the zero-load magnetic flux was obtained by neglecting the dead weight. The magnetic flux of the object was measured after the loading was stable at all levels. The magnetic flux in the results of the magnetic effect system in Figure 12 was compared with the flux calibrated in the same environment.

In the initial loading stage, the overall change of the steel truss model structure was not significant; the working performance of each member was optimal. The force was uniform and reasonable without obvious deformation. When the applied external force increased gradually, the measured points of plane A–B sunk slightly and the bottom of the model bent slightly. This shows that the bending moment at the bottom of the steel truss model structure increases gradually, the deformation increases, and the stress of each member increases continuously. As shown in Figure 12, the magnetic flux measurement system based on the existing magnetism of the rods is in good agreement with the traditional strain method. In particular, a special inflection point appears in Figure 12d, which shows the load–stress relationship of the 3–9 member in the truss structure. When the load on the 3–9 member gradually increases from 0, its stress increases slowly, which is a small tensile stress. When the applied load reaches approximately 75 N, compressive stress begin to appear in the member and increase approximately linearly with the increasing load. Therefore, an inflection point appears in Figure 12d. By measuring the magnetic flux variation of the existing magnetism of the steel truss model, the constitutive magnetic stress flux model presented in this paper can better reflect the actual stress variation of the steel truss model structural members.

Compared with the traditional stress–strain method, the magnetic stress testing system can ignore the loading history of structural members and the constitutive model of structural member materials. Through the development of reasonable testing probe, the stress of structural members can be tested nondestructively online using the magnetic stress testing system.

To further verify the validity and correctness of the existing constitutive magnetic stress flux model, the finite element software ANSYS was used to establish the simulation calculation model of the steel truss structure. The stress changes of the upper chord, the web, and the lower chord under the action of force were simulated and calculated. The results for the stress states under the action of force were obtained. Compared with the experimental results, the validity of the magnetic flux stress measurement method based on the existing magnetism was verified.

The finite element calculation model was established according to the size and specification of steel truss structure model designed in this paper. Beam unit 188 was used to simulate each member of the steel truss component in finite element model. In the numerical simulation, the material used in the model was Q420qD, the modulus of elasticity was 2×10^11^ Pa, the Poisson’s ratio was 0.3, and the density was 7850 kg/m³.

In the simulation calculation, the loading started from zero and went up to 400 N in increments of 20 N. After each loading, the standing of corresponding time interval was set according to the regulations. ANSYS software simulates the actual loading process by using the multiload step static solution method, which is divided into 18 load steps to complete the whole loading process. In this paper, the overall force under 400 N test loading of the steel truss components and the force in previous test rod were analyzed.

Figure 12 shows the load–stress curves of the AB plane of the steel truss model structure in the same positions in the middle of sections 1–2, 1–7, 3–4, 3–9, 4–10, 6–7, 8–9, and 10–11 from the magnetic flux stress measurement system, strain measurement, and simulation calculation based on the existing magnetism.

Through the simulation and calculation of the finite element model, the numerical test results were extracted and compared with the test data obtained using the magnetic flux measurement system and the traditional strain measurement in the static loading model test. The stress test results of the steel truss corresponding to the rod model test were close to the numerical simulation results and were consistent with the actual stress state of the corresponding members tested by the steel truss model.

## 4. Discussion

In the test, the probe of the magnetic stress testing system we developed was suitable for components with a circular cross-section, and had good applicability in the nondestructive stress detection of rod-type structural components. For example, satisfactory stress detection was achieved for the members of the truss structure, the cables of the long span structure, the hangers, and the mechanical two-force members. In actual engineering, some components are in inaccessible positions. Data transmission via a magnetic fluxmeter is a technical problem. On the theoretical basis of this paper, the integrated magnetic stress induction system using wireless data transmission technology is an important research direction.

During the service period of the structure, the mechanically connected tensile and compressive members can be disassembled during the test and a magnetic stress induction system can be installed to achieve real-time stress detection of the structure in service. The stresses of structural members in service are more complicated. Therefore, in this study, stress tests were carried out for the ideal tensile and compressive stress of the structural steel specimen (Figure 7) and the tensile and compressive members in the truss structure (Figure 10). The theoretical magnetic stress model obtained by the experiment was the theoretical basis for the further study of the probe, which can be applied to components with different cross-sectional shapes and forced forms. According to the research method in this paper, the stress detection of structural members under complex load can be achieved. The stress test studied in this paper and the mechanical test are comparable. It can also be said that stress testing is a very important aspect of mechanical testing. The test results of the magnetic stress testing system can directly assess the stress of structural members.

## 5. Conclusions

Through the stress detection testing of steel truss members using a magnetic stress sensing system based on the existing magnetic flux method, the following conclusions were drawn:

1. The magnetic stress sensing system, which integrates a magnetic flux induction coil, a magnetic flux measurement device, a loaded device, and data acquisition software, can be used for nondestructive online testing of the stress of structural members;
2. The existing magnetism of the structural bridge and building steels is affected by the chemical composition, grade, and diameter. The effect of the existing magnetism on the initial magnetization is obvious. The maximum magnetization in the magnetization curve is different. The maximum magnetization of the structural bridge steel is higher than that of structural building steel of the same grade;
3. In the range of linear elasticity, the magnetic flux variation of structural steel specimens based on the existing magnetism is related to the steel grade and diameter of specimens, which increases with the increase of the stress. By using the magnetic stress flux mathematical model based on the existing magnetism, nondestructive online detection of the stress of structural members can be realized by measuring the magnetic flux;
4. The stress detection of the steel truss model structure was carried out by measuring the magnetic flux using the magnetic stress sensing system. This method can overcome the deficiency of the loading history required by traditional strain measurements. The test results were in good agreement with the stress–strain constitutive relation;
5. The results of the magnetic flux measurement by the magnetic stress sensing system based on the existing magnetism of the steel truss structure agree well with those of the numerical simulation and calculation. The validity of the magnetic stress flux mathematical model based on existing magnetism established in this paper is verified.

## Figures and Tables

**Table 1:** Quality composition and mechanical properties of structural steel samples.

Type	Grade	Chemical Composition (%)								
		C	Si	Mn	P	S	V	Nb	Ti	Al
Structural building steel	Q235B	0.15	0.20	0.38	0.040	0.024	—	—	—	—
	Q345B	0.15	0.45	1.30	0.020	0.035	0.08	0.040	0.10	—
	Q390B	0.15	0.45	1.30	0.020	0.035	0.10	0.040	0.10	—
	Q420B	0.13	0.35	1.30	0.025	0.030	0.15	0.035	0.15	—
Structural bridge steel	Q235qD	0.17	0.25	0.68	0.016	0.015	—	—	—	0.017
	Q345qD	0.16	0.41	1.35	0.016	0.015	—	—	—	0.016
	Q375qD	0.16	0.41	1.35	0.016	0.015	—	—	—	0.016
	Q420qD	0.16	0.45	1.50	0.016	0.010	—	—	—	0.016

**Table 2:** Maximum magnetization intensity values of specimens with different grades.

Grades	Maximum Magnetization (A·m^2^)	Grades	Maximum Magnetization (A·m^2^)
Q235B	1.72	Q235qD	1.78
Q345B	1.75	Q345qD	1.81
Q390B	1.79	Q375qD	1.84
Q420B	1.83	Q420qD	1.87

**Table 3:** The magnetic flux fitting equation for steel specimens with a diameter of 32 mm.

Specimens	Direction	Fitting Equation
Q235B	forward	y = −9 × 10^−5^x^2^ + 0.0023x + 8.1683
	reverse	y = 7 × 10^−5^x^2^ + 0.0007x − 5.998
Q345B	forward	y = −8 × 10^−5^x^2^ + 0.0019x + 6.8152
	reverse	y = 9 × 10^−5^x^2^ − 0.0008x − 5.8377
Q390B	forward	y = −5 × 10^−5^x^2^ + 0.0012x + 4.0601
	reverse	y = 5 × 10^−5^x^2^ − 0.0012x − 4.0601
Q420B	forward	y = −2 × 10^−5^x^2^ − 0.0002x + 1.2025
	reverse	y = 2 × 10^−5^x^2^ + 0.0002x − 1.2025
Q235qD	forward	y = −7 × 10^−5^x^2^ − 0.0016x + 6.0111
	reverse	y = 6 × 10^−5^x^2^ −0.0016x − 4.7525
Q345qD	forward	y = −7 × 10^−5^x^2^ − 6 × 10^−5^x + 5.167
	reverse	y = 5 × 10^−5^x^2^ − 0.0008x − 4.1536
Q375qD	forward	y = −7 × 10^−5^x^2^ − 0.0006x + 5.4675
	reverse	y = 6 × 10^−5^x^2^ − 4 × 10^−5^x − 4.4287
Q420qD	forward	y= −8 × 10^−5^x^2^ + 0.0015x + 5.6011
	reverse	y = 7 × 10^−5^x^2^ − 0.0013x − 4.5002
